# Automated volumetric modulated arc therapy planning for whole pelvic prostate radiotherapy

**DOI:** 10.1007/s00066-017-1246-2

**Published:** 2017-12-21

**Authors:** Martin Buschmann, Abdul Wahab M. Sharfo, Joan Penninkhof, Yvette Seppenwoolde, Gregor Goldner, Dietmar Georg, Sebastiaan Breedveld, Ben J. M. Heijmen

**Affiliations:** 1 Department of Radiation Oncology, Medical University of Vienna/AKH Wien, Währinger Gürtel 18-20, 1090 Vienna, Austria; 2 Christian Doppler Laboratory for Medical Radiation Research for Radiation Oncology, Medical University of Vienna, Vienna, Austria; 3 Department of Radiation Oncology, Erasmus MC Cancer Institute, Rotterdam, The Netherlands

**Keywords:** Prostate cancer, Lymph nodes, Volumetric modulated arc therapy, Organs at risk, Multicriteria optimization, Prostatakrebs, Lymphknoten, Volumenmodulierte Arc-Therapie, Risikoorgane, Multikriterielle Optimierung

## Abstract

**Background:**

For several tumor entities, automated treatment planning has improved plan quality and planning efficiency, and may enable adaptive treatment approaches. Whole-pelvic prostate radiotherapy (WPRT) involves large concave target volumes, which present a challenge for volumetric arc therapy (VMAT) optimization. This study evaluates automated VMAT planning for WPRT-VMAT and compares the results with manual expert planning.

**Methods:**

A system for fully automated multi-criterial plan generation was configured for each step of sequential-boost WPRT-VMAT, with final “autoVMAT” plans being automatically calculated by the Monaco treatment planning system (TPS; Elekta AB, Stockholm, Sweden). Configuration was based on manually generated VMAT plans (manualVMAT) of 5 test patients, the planning protocol, and discussions with the treating physician on wishes for plan improvements. AutoVMAT plans were then generated for another 30 evaluation patients and compared to manualVMAT plans. For all 35 patients, manualVMAT plans were optimized by expert planners using the Monaco TPS.

**Results:**

AutoVMAT plans exhibited strongly improved organ sparing and higher conformity compared to manualVMAT. On average, mean doses (D_mean_) of bladder and rectum were reduced by 10.7 and 4.5 Gy, respectively, by autoVMAT. Prostate target coverage (V_95%_) was slightly higher (+0.6%) with manualVMAT. In a blinded scoring session, the radiation oncologist preferred autoVMAT plans to manualVMAT plans for 27/30 patients. All treatment plans were considered clinically acceptable. The workload per patient was reduced by > 70 min.

**Conclusion:**

Automated VMAT planning for complex WPRT dose distributions is feasible and creates treatment plans that are generally dosimetrically superior to manually optimized plans.

**Electronic supplementary material:**

The online version of this article (10.1007/s00066-017-1246-2) contains supplementary material, which is available to authorized users.

## Zusammenfassung

**Hintergrund:**

Automatisierte Bestrahlungsplanung zeigte bei einigen Tumorentitäten Vorteile durch verbesserte Planqualität und höhere Planungseffizienz, was die Einführung adaptiver Bestrahlungstechniken erleichtern könnte. Die Ganzbecken-Prostatabestrahlung (WPRT) beinhaltet große konkave Zielvolumina und stellt eine Herausforderung für die Optimierung der volumenmodulierten Arc-Therapie (VMAT) dar. Die Studie evaluiert die automatisierte VMAT-Planung für WPRT und vergleicht die Ergebnisse mit der manuellen Planung.

**Methoden:**

Ein System für die vollautomatisierte multikriterielle Planung wurde für die WPRT mit sequenziellem Boost konfiguriert, wobei die finalen AutoVMAT-Pläne im Monaco-Planungssystem (Elekta AB, Stockholm, Schweden) berechnet wurden. Die Konfiguration basierte auf manuell erstellten VMAT-Plänen (ManualVMAT) von 5 Testpatienten, dem klinischen Planungsprotokoll und Diskussionen mit dem behandelten Arzt zu Planverbesserungen. AutoVMAT-Pläne wurden für weitere 30 Evaluierungspatienten erstellt und mit ManualVMAT-Plänen verglichen. Die ManualVMAT-Pläne wurden für alle 35 Patienten von erfahrenen Planern in Monaco optimiert.

**Ergebnisse:**

AutoVMAT-Pläne wiesen stark verbesserte Organschonung und höhere Konformität im Vergleich zu ManualVMAT-Plänen auf. Die mittlere Dosis (D_mean_) von Harnblase und Rektum wurden im Durchschnitt um 10,7 und 4,5 Gy mit AutoVMAT reduziert. Die Zielgebietsabdeckung der Prostata (V_95%)_ war für ManualVMAT etwas höher (+0,6 %). In einer Blindbewertung bevorzugte der behandelnde Radioonkologe den AutoVMAT-Plan gegenüber dem ManualVMAT-Plan bei 27/30 Patienten. Alle Bestrahlungspläne wurden als klinisch akzeptabel bewertet. Der manuelle Arbeitsaufwand pro Patient reduzierte sich um > 70 min.

**Schlussfolgerung:**

Die automatisierte VMAT-Planung ist für komplexe Dosisverteilungen bei WPRT durchführbar und generiert im Allgemeinen Bestrahlungspläne, die den manuell optimierten Plänen dosimetrisch überlegen sind.

Modern linear accelerator (linac)-based prostate radiotherapy (RT) is commonly delivered by intensity-modulated radiation therapy (IMRT) and, more recently, by volumetric modulated arc therapy (VMAT). However, treatment planning is often an iterative trial-and-error process, and the resulting plan quality is strongly dependent on the planner’s experience.

The clinical target volume (CTV) usually comprises the prostate gland, but in intermediate- and high-risk disease, the seminal vesicles and pelvic lymph nodes are also commonly irradiated. This technique of whole-pelvic prostate RT (WPRT) has shown clinical benefit compared to local prostate-only RT in several studies [1, 2], but its role remains controversial [3, 4]. The optimization of WPRT-IMRT/VMAT plans is more challenging and time consuming than for the local prostate treatments, due to the larger and more complex target volumes.

In recent years, multi-criterial optimization (MCO) has been applied in the field of treatment plan optimization with the aim of generating Pareto-optimal plans which cannot be improved further in one aspect without worsening another. Another motivation for advanced optimization is avoidance of time-consuming iterative treatment planning, which may still lead to treatments lacking Pareto optimality. An MCO approach developed by Craft et al. [5, 6] uses manual navigation of a plan library that spans the Pareto space. This strategy can be classified as an a posteriori technique, where manual interaction by the user is necessary to select the best-fitting treatment plan. Erasmus-iCycle is a multi-criterial optimizer for beam profiles and beam angle selection [7] which employs an a priori strategy. The user defines a treatment site-specific optimization protocol, a so-called wishlist, containing the goal functions that are optimized in a specific order, defined by assigned priorities. The priorities steer the fully automated multi-criterial plan generation. In addition, the wishlist can contain hard constraints that must not be violated. Generated plans are Pareto optimal and clinically favorable [8–10]. Currently, Erasmus-iCycle is used as a pre-optimizer; optimized iCycle plans are subsequently reconstructed with Monaco treatment planning system (TPS; Elekta AB, Stockholm, Sweden) to generate deliverable IMRT or VMAT plans [9, 11]. After the development of a wishlist and a translation strategy to the TPS in a multidisciplinary team, treatment planning with Erasmus-iCycle/Monaco is fully automatic. The algorithm is successfully applied in clinical routine for several tumor sites, including head and neck [8], cervix [12], lung [10], spinal metastases [13], and local prostate cancer [9]. Up until now, the algorithm has only been validated against manual planning at a single institution, and the previously demonstrated benefit of autoplanning may be explainable by institution-specific protocols.

This study investigates the feasibility of automated generation of VMAT plans for WPRT with Erasmus-iCycle/Monaco and compares the resulting dose distributions with manually optimized VMAT plans that were generated by expert planners in a different center, also using Monaco.

## Materials and methods

### Patients

From the clinical database of the department of Radiation Oncology in Vienna, 35 prostate cancer patients who were treated with WPRT-VMAT between May 2014 and July 2016 were randomly selected for this study. Patients with hip implants and bladder filling less than 100 ml on the planning CT scan were not included. Each patient had gold fiducials implanted in the prostate for improved target localization during image guidance. A CT scan with 2 mm slice thickness was used for treatment planning. Patients were scanned and treated with a rectal balloon. The prostate was delineated as the primary CTV (CTV-P), and the pelvic lymph nodes including seminal vesicles as well as the CTV-P were defined as the secondary CTV (CTV-LN). Rectum, bladder, bowel bag, and femoral heads were manually contoured as organs at risk (OAR). The bowel was delineated up to a 2 cm extension of the target in the cranial direction. Considering the use of daily image guidance, setup margins of 5 mm were applied to create two planning target volumes: PTV-P and PTV-LN.

The total radiation dose was prescribed in a two-phase Vienna-specific protocol: the first phase delivered 60 and 50 Gy in 25 fractions to PTV-P and PTV-LN, respectively, using a simultaneous integrated boost (SIB) technique. The second phase was a sequential boost of 13 Gy in 5 fractions to PTV-P. Clinical acceptability of the treatment plans was assessed through a list of clinically applied organ dose constraints. which is presented in Table 1, and by clinical judgment of the treating physician.

**Table 1:** Clinical dose constraints for organs at risk for the total summed dose

Rectum	D_max_	78 Gy
	V_65Gy_	20%
	V_60Gy_	40%
	V_55Gy_	45%
	V_50Gy_	50%
Bowel bag	D_max_	56 Gy
	V_50Gy_	10%
	V_45Gy_	15%
	V_40Gy_	20%
Bladder	D_max_	78 Gy
	V_65Gy_	20%
	V_55Gy_	40%
	V_50Gy_	50%
	V_35Gy_	80%
Femoral heads	D_max_	55 Gy
	V_45Gy_	5%

*D*_*max*_ maximum dose, *V*_*xGy*_ relative volume of organ receiving at least x Gy

### Treatment planning and delivery systems

Prior to initiation of this study, discussions between the radiation oncologist (GG), the dosimetrist, and the physicists (MB, AS, YS, JP, BH) were held to define the guidelines and preferences for dose distributions. A target dose criterion was defined as a minimum target coverage corresponding to total volume irradiated with 95% of the prescribed dose (V_95%_) = 96% for PTV-P and PTV-LN in the total summed dose (phase 1 plus phase 2). The physician expressed his preference for high target coverage and therefore the aim was to reach approximately V_95%_ = 98% during optimization of the two single treatment plans.

All final autoVMAT and manualVMAT plans were generated with the Monaco TPS version 5.11 for a VersaHD linac (Elekta) with 10 MV photons. All treatments were planned to be delivered by full 360° coplanar arcs. Phase 1 was planned as dual arc and the boost phase 2 as single arc, with the isocenter being placed in the center of PTV-LN and PTV-P, respectively.

### Manual VMAT planning

Two experienced planners from the Vienna team performed VMAT treatment planning (manualVMAT) in Monaco for all 35 patients according to current clinical guidelines, with commonly used templates of optimization cost functions. The employed templates are described in the supplementary material. For each patient, the optimization parameters were iteratively tweaked to improve the dose distributions. This process was performed in the absence of time constraints and the planner only stopped when no further improvement in plan quality could be achieved. The two plan phases were optimized and finalized independently before the summed dose was inspected to check the OAR constraints. Each patient was planned by just one planner.

### Automated VMAT planning with Erasmus-iCycle/Monaco

For automated VMAT planning (autoVMAT), the Erasmus-iCycle algorithm [7] was used as a pre-optimizer. To this purpose, a VMAT plan was approached by using 20 equi-angularly positioned IMRT beams. The two wishlists for the two treatment phases used in this study are presented in Table 2 and a detailed description is provided in the supplementary material. The segmentation settings in Monaco were identical for manual and automated plan generation, and are described in the supplementary material.

**Table 2:** Applied wishlists for automatic volumetric modulated arc therapy plan generation for (A) phase 1 (i. e., whole pelvis) and (B) phase 2 (i. e., boost) plans. A detailed description is included in the supplementary material. The parameters k and α are specific to the iCycle algorithm [7]

**(A) Constraints**				
*Volume*		*Type*	*Limit*	
PTV‑P		Max	105% of D_high_	
PTV‑P		Mean	101% of D_high_	
PTV-LN − PTV‑P_+5mm_		Max	105% of D_low_	
PTV-LN − PTV‑P_+5mm_		Mean	101% of D_low_	
Left + right femoral head		Max	70% of D_high_	
PTV-LN shell 50 mm		Max	50% of D_high_	
Unspecified tissues		Max	105% of D_high_	
**Objectives**				
*Priority*	*Volume*	*Type*	*Goal*	*Parameters*
1	PTV‑P	↓ LTCP	0.65	D_high_ = 60 Gy, α = 0.7
2	PTV-LN	↓ LTCP	0.5	D_low_ = 50 Gy, α = 0.9
3	PTV-LN shell 5 mm	↓ Max	90% of D_low_	–
4	Rectum	↓ EUD	70% of D_high_	k = 12
	Rectum	↓ EUD	30% of D_high_	k = 4
5	Bladder	↓ EUD	30% of D_high_	k = 4
6	PTV-LN shell 15 mm	↓ Max	60% of D_low_	–
	PTV-LN shell 25 mm	↓ Max	40% of D_low_	–
7	Bowel bag	↓ EUD	30% of D_high_	k = 8
8	Skin ring 20 mm	↓ Max	30% of D_high_	–
9	Rectum	↓ Mean	20% of D_low_	–
10	Bladder	↓ Mean	20% of D_low_	–
11	Bowel bag	↓ Mean	20% of D_low_	–
12	Left + right femoral head	↓ EUD	10% of D_high_	k = 5
**(B) Constraints**				
*Volume*		*Type*	*Limit*	
PTV‑P		Max	105% of D^p^	
PTV‑P		Mean	101% of D^p^	
Left + right femoral head		Max	70% of D^p^	
PTV‑P shell 50 mm		Max	50% of D^p^	
Unspecified tissues		Max	105% of D^p^	
**Objectives**				
*Priority*	*Volume*	*Type*	*Goal*	*Parameters*
1	PTV‑P	↓ LTCP	0.5	D^p^ = 13 Gy, α = 0.95
2	PTV‑P shell 5 mm	↓ Max	90% of D^p^	–
3	Rectum	↓ EUD	70% of D^p^	k = 16
4	Bladder	↓ EUD	60% of D^p^	k = 12
5	PTV‑P shell 15 mm	↓ Max	50% of D^p^	–
	PTV‑P shell 25 mm	↓ Max	30% of D^p^	–
	Skin ring 20 mm	↓ Max	30% of D^p^	–
6	Rectum	↓ Mean	10% of D^p^	–
7	Bladder	↓ Mean	10% of D^p^	–
8	Left + right femoral head	↓ EUD	10% of D^p^	k = 5

*PTV‑P* planning target volume of the prostate, *D* ^*p*^ prescribed dose, *LTCP* logarithmic tumor control probability, *EUD* equivalent uniform dose, *α* cell sensitivity, *k* volume effect, *PTV-LN* planning target volume of the lymph nodes, *D*_*high*_ high (=60 Gy) prescribed dose, *D*_*low*_ low (=50 Gy) prescribed dose, *PTV‑P*_*+5mm*_ a 5 mm transition region within PTV_LN_

The autoVMAT strategy was developed using a random subset of 5 of the total of 35 study patients. This training cohort was used to configure the Erasmus-iCycle/Monaco system for high-quality automated VMAT plan generation, i. e., development of the site-specific wishlists and automatic translations of pre-optimization results into patient-specific Monaco templates for both treatment phases. A first strategy for autoVMAT was created according to clinical guidelines and applied on the training cohort to create deliverable VMAT plans. A “flavor session” with the radiation oncologist and medical physicists was then held, where manualVMAT and autoVMAT plans for the training patients were blindly presented and discussed. Using the results and physicians’ preferences from this flavor session, the autoVMAT configurations were refined and then applied to the 30 patients not used for configuration (evaluation patients).

### Dosimetric plan evaluations

For the 30 evaluation patients, the dose distributions of the individual treatment phases and the summed total dose as generated by autoVMAT and manualVMAT were analyzed and compared. Normal tissue sparing was assessed by extracting the near-maximum dose D_2%_ and mean dose (D_mean_) for each OAR, and the total volume irradiated at 95 and 50% of the respective prescription dose. Additionally, for the rectum, V_70Gy_ and V_55Gy_ were assessed in the summed dose, as these parameters are correlated to rectal toxicity [14] (adjusted for hypofractionation according to the linear quadratic model with α/β = 3). For target structures, V_95%_ (target coverage), D_2%_, and D_mean_ were analyzed. The homogeneity (HI) and two conformity indices (CI, CI_50%_) were calculated as follows:

$$HI=(D_{2\mathrm{\% }}-D_{98\mathrm{\% }})/D_{50\mathrm{\% }},$$

$$CI=V_{95\% }/V_{\text{PTV}},$$

$$Cl_{50\% }=V_{50\% }/V_{\text{PTV}},$$

where V_95%_ and V_50%_ describe the total volumes irradiated with 95 and 50% of the prescribed dose, respectively. For the determination of D_2%_, D_mean_, and HI in the PTV-LN, the SIB volume including a 2 cm margin was subtracted.

Dosimetric differences between autoVMAT and manualVMAT were tested for statistical significance by a two-sided Wilcoxon matched-paired signed-rank test (*p* < 0.05). No rescaling of dose distributions was performed, since D_mean_ was equivalent in the total plan sum for autoVMAT and manualVMAT. The number of monitor units (MU) for each of the two treatment phases was noted.

### Physician’s plan scoring

In a blinded plan scoring session, the radiation oncologist was simultaneously presented with the total summed plans created by autoVMAT and manualVMAT for all 30 study patients. The cumulative DVHs for all structures, the dose distributions in the TPS, and a table with the most important dosimetric parameters were provided to the physician. Based on this information, the physician rated the two distributions relatively to each other according to three categories:

- Plan A is considerably better than plan B
- Plan B is considerably better than plan A
- Plan A and plan B are of similar quality

### Dosimetric verification measurements

Deliverability of autoVMAT and manualVMAT plans was checked by performing verification dose measurements for 10 randomly selected patients. Dose distributions were measured with the Delta^4^ phantom (ScandiDos, Uppsala, Sweden) and compared to planned doses through a gamma analysis (3% local dose difference, 3 mm).

## Results

### Dosimetric plan evaluations

**Fig. 1:**
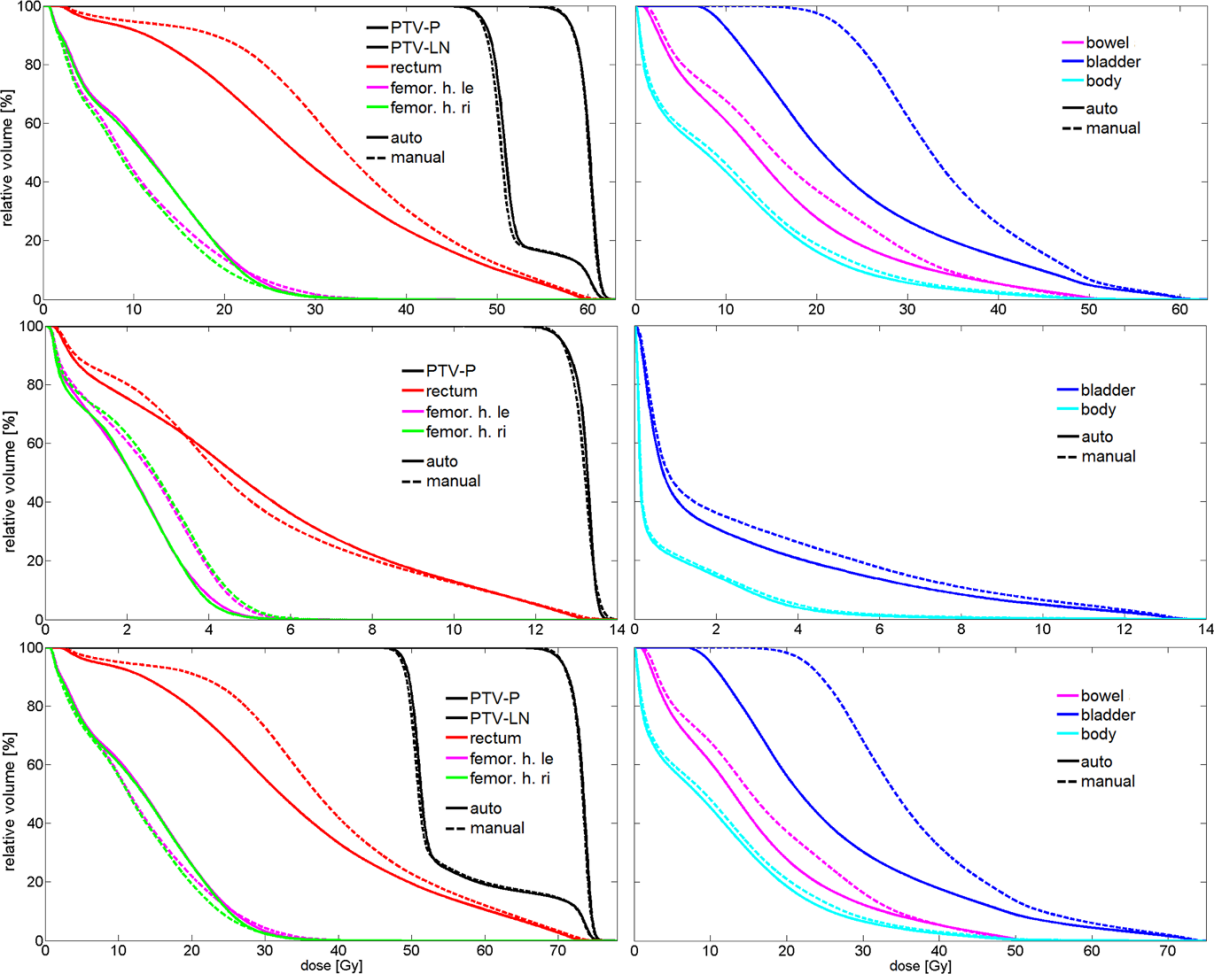
Mean dose–volume histograms (DVHs) for automated and manual volumetric modulated arc therapy (autoVMAT, manualVMAT) plans for the 30 evaluation patients. *Top*: phase 1 whole-pelvis plan; *middle*: phase 2 boost plan; *bottom*: sum plan; *femor. h.* femoral head. *PTV* *-P* planning target volume prostate,* PTV-LN* planning target volume lymph nodes

All generated autoVMAT and manualVMAT dose distributions fulfilled the criteria for clinical acceptability in terms of OAR sparing and target coverage. Population average DVHs for the 30 evaluation patients for the single plan phases and the total treatment are shown in Fig. 1, pointing toward meaningful advantages for autoVMAT. Dose–volume parameters of autoVMAT and differences with manualVMAT are presented in Table 3.

**Table 3:** Dosimetric parameters of autoVMAT plans and differences to manualVMAT plans

	Phase 1 whole pelvis plan			Phase 2 boost plan			Total dose		
	AutoVMAT	ManualVMAT-autoVMAT	*p*-value	AutoVMAT	ManualVMAT-autoVMAT	*p*-value	AutoVMAT	ManualVMAT-autoVMAT	*p*-value
	Mean ± std	Mean ± std		Mean ± std	Mean ± std		Mean ± std	Mean ± std	
CI	1.16 ± 0.04	0.05 ± 0.05	<0.001	1.10 ± 0.05	0.07 ± 0.05	<0.001	–	–	–
CI_50%_	4.50 ± 0.35	1.17 ± 0.39	<0.001	3.93 ± 0.35	0.64 ± 0.24	<0.001	–	–	–
CI SIB	1.10 ± 0.03	0.02 ± 0.04	0.026	–	–	–	–	–	–
HI	0.10 ± 0.01	−0.01 ± 0.01	<0.001	0.09 ± 0.01	0.00 ± 0.01	NS	–	–	–
HI SIB	0.08 ± 0.01	−0.01 ± 0.01	0.003	–	–	–	–	–	–
PTV‑P V_95%_ (%)	97.56 ± 0.76	0.49 ± 0.93	0.023	98.27 ± 0.57	0.50 ± 0.48	<0.001	97.75 ± 0.61	0.60 ± 0.77	<0.001
PTV‑P D_2%_ (Gy)	61.73 ± 0.11	−0.11 ± 0.28	0.039	13.60 ± 0.02	0.08 ± 0.08	<0.001	75.06 ± 0.11	−0.12 ± 0.28	0.020
PTV‑P D_mean_ (Gy)	59.93 ± 0.12	−0.05 ± 0.25	NS	13.16 ± 0.03	−0.04 ± 0.06	<0.001	73.08 ± 0.13	−0.09 ± 0.27	NS
PTV-LN V_95%_ (%)	98.14 ± 0.70	−0.28 ± 0.84	NS	–	–	–	99.30 ± 0.31	−0.36 ± 0.60	0.003
PTV-LN D_2%_ (Gy)	52.38 ± 0.20	−0.56 ± 0.28	<0.001	–	–	–	54.42 ± 1.26	−0.18 ± 0.59	NS
PTV-LN D_mean_ (Gy)	50.33 ± 0.18	−0.41 ± 0.20	<0.001	–	–	–	50.77 ± 0.19	−0.35 ± 0.21	<0.001
Rectum D_2%_ (Gy)	57.58 ± 1.34	0.90 ± 0.75	<0.001	12.66 ± 0.30	0.15 ± 0.12	<0.001	70.17 ± 1.63	1.05 ± 0.80	<0.001
Rectum D_mean_ (Gy)	29.18 ± 3.63	4.63 ± 2.08	<0.001	5.05 ± 0.84	−0.08 ± 0.36	NS	34.23 ± 4.00	4.53 ± 2.16	<0.001
Rectum V_70Gy_ (%)	–	–	–	–	–	–	2.68 ± 1.58	0.60 ± 0.57	<0.001
Rectum V_55Gy_ (%)	–	–	–	–	–	–	14.59 ± 4.83	2.22 ± 1.76	<0.001
Bladder D_2%_ (Gy)	55.30 ± 4.01	1.40 ± 1.47	<0.001	11.72 ± 1.33	0.69 ± 0.72	<0.001	66.67 ± 5.76	2.25 ± 2.18	<0.001
Bladder D_mean_ (Gy)	24.03 ± 3.73	10.25 ± 3.03	<0.001	2.20 ± 1.07	0.44 ± 0.31	<0.001	26.28 ± 4.18	10.68 ± 3.21	<0.001
Hip left D_2%_ (Gy)	26.38 ± 3.28	2.14 ± 3.33	0.002	4.46 ± 0.55	0.56 ± 0.43	<0.001	30.00 ± 3.70	2.21 ± 3.23	<0.001
Hip left D_mean_ (Gy)	11.53 ± 1.38	−1.22 ± 1.28	<0.001	1.98 ± 0.36	0.40 ± 0.26	<0.001	13.52 ± 1.63	−0.83 ± 1.32	0.003
Hip right D_2%_ (Gy)	26.01 ± 3.38	0.18 ± 3.13	NS	4.39 ± 0.47	0.83 ± 0.58	<0.001	29.54 ± 3.45	0.20 ± 3.11	NS
Hip right D_mean_ (Gy)	11.32 ± 1.25	−1.64 ± 1.05	<0.001	1.95 ± 0.30	0.50 ± 0.22	<0.001	13.29 ± 1.44	−1.15 ± 1.16	<0.001
Bowel D_2%_ (Gy)	45.17 ± 3.29	0.26 ± 1.73	NS	–	–	–	45.30 ± 3.28	0.26 ± 1.73	NS
Bowel D_mean_ (Gy)	15.22 ± 3.29	2.07 ± 1.24	<0.001	–	–	–	15.27 ± 3.30	2.09 ± 1.24	<0.001
MU	1029 ± 130	−365 ± 115	<0.001	716 ± 89	−153 ± 70	<0.001	–	–	–

*autoVMAT* automated volumetric modulated arc plan, *manualVMAT* manual volumetric modulated arc plan, *NS* non-significant, *PTV‑P* planning target volume of the prostate, *PTV-LN* planning target volume of the lymph nodes, *CI* conformity index, *HI* homogeneity index, *SIB* simultaneous integrated boost, *V*_*95%*_ volume receiving at least 95% of the prescribed dose, *D*_*mean*_ mean dose, *D*_*2%*_ minimum dose delivered to the most exposed 2% of the strucutre, *V*_*70 Gy*_ and *V*_*55 Gy*_ are the volumes receiving 70 Gy, respectively 55 Gy, or more, *MU* monitur units, *std* standard deviation

**Fig. 2:**
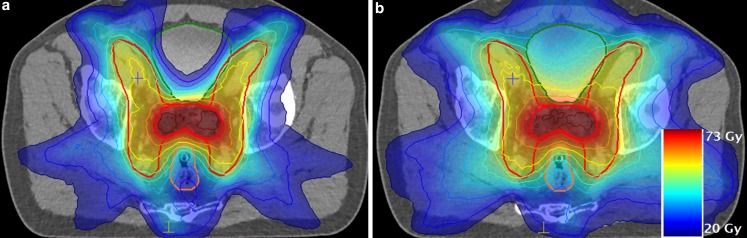
Exemplary dose distribution of the total summed plan. **a** Automated volumetric modulated arc plan (autoVMAT); **b** manual VMAT plan (manualVMAT). Most striking is the improved bladder sparing with autoVMAT, which was also the most important advantage of autoVMAT for the entire study population

Fig. 2 depicts an exemplary dose distribution in an axial slice of the summed treatment for both optimization techniques.

In general, dose distributions in the targets were similar, with small differences as is shown by the DVHs and Table 3. D_mean_ in PTV-P was identical in phase 1 and the summed dose in both optimizations. Target coverage was similar for both optimization methods, but was statistically significantly higher in manualVMAT in the PTV-P by a small amount of 0.6% points on average. In the PTV-LN, V_95%_ was higher in autoVMAT in the summed plan. Significantly lower OAR doses were observed in rectum, bladder, and bowel in autoVMAT compared to manualVMAT, for both phases and for the sum plan. Most prominent was the lower bladder dose in autoVMAT, with average D_mean_ values in the sum plan of 26.3 and 37 Gy for autoVMAT and manualVMAT, respectively. Doses to the femoral heads were generally higher in autoVMAT, but the average difference in D_mean_ was below 1.7 Gy.

AutoVMAT dose distributions were more conformal than manualVMAT doses, which was especially evident in the smaller CI_50%_ in both planning phases. The volume of the 50% isodose was decreased on average by 852 ml in phase 1 (50% = 25 Gy) and 63 ml in phase 2 (50% = 6.5 Gy) with autoVMAT. ManualVMAT plans delivered slightly more homogeneous target doses. The average number of MUs were 664/1029 for phase 1 and 563/716 for phase 2 in manualVMAT and autoVMAT, respectively.

### Physician’s plan scoring

During the blinded scoring session, the radiation oncologist considered all plans from both optimizations to be clinically acceptable. He preferred the autoVMAT plan in 27 out of 30 patients and the manualVMAT in the other 3 patients. He chose the autoVMAT plans mainly for their increased OAR sparing, while the occasional small advantages of manualVMAT in PTV-P coverage were not considered clinically relevant in most cases. However, in the 3 patients for whom manualVMAT was considered better, the V_95%_ and D_min_ were noticeably lower in autoVMAT. After the scoring session, the MUs of the treatment plans in these 3 patients were rescaled to reach identical values of V_95%_ in the PTV-P in the autoVMAT and manualVMAT plans, and another blinded scoring session was held with these 3 patients. The physician then preferred the autoVMAT plans in the second round in all cases.

### Planning workload

The planning workload in manualVMAT was assessed by recording the time for parameter tweaking and reoptimization after the initial optimization run, as the planner would usually not wait in front of the workstation during the first calculation. The average manual planning time was 54 min for a phase 1 plan and 22 min for a phase 2 plan, which accumulated to 76 min workload per patient. The additional optimization time in Erasmus-iCycle for autoVMAT was in the range of 3–6h for phase 1 and 20–40 min for phase 2.

### Dosimetric verification measurements

All treatment plans, both autoVMAT and manualVMAT, exhibited gamma pass rates (GPR) >95% in the verification measurements, fulfilling the in-house QA requirements. The mean GPR in phase 1/phase 2 were 98.3%/99.3% for autoVMAT and 98.6%/99.6% for manualVMAT. Differences between the verification results of both optimization methods were not statistically significant.

## Discussion

In this study, the potential of fully automated, a priori multi-criterial VMAT planning for whole-pelvis prostate treatments was investigated for the first time. This treatment site involves a large concave-shaped target with a SIB, and therefore presents a new and complex optimization problem for the algorithm.

Automated planning significantly improved OAR sparing compared to manual planning. In the total sum plans, the dose to the bladder, rectum, and bowel was substantially reduced. Although a slightly higher target coverage in the prostate PTV was observed in manualVMAT, this small difference was not considered clinically relevant. Additionally, the conformity strongly improved with autoVMAT. The dosimetric advantages were also confirmed by the radiation oncologist, who preferred the autoVMAT plan over manualVMAT in 90% of cases in a blinded plan comparison session.

The driving forces of autoplanning with Erasmus-iCycle are the wishlists, which essentially define planning protocols or recipes to be followed by the algorithm for automatic plan generation The wishlists are specific for a patient population. In contrast to generally applied planning templates, the wishlists do not need patient-specific fine-tuning. Wishlists are generated in an iterative procedure, using repeated automated plan generations for a small group of test patients.

AutoVMAT planning in this study was indeed fully automatic with no hands-on time, resulting in a reduction of more than 70 min of manual planning time. However, the creation and refining of wishlists in a multidisciplinary team prior to autoplanning for a patient population is time-consuming, which needs to be considered in the departmental logistics.

The number of MUs was significantly higher in autoVMAT than in manualVMAT plans, as a result of higher modulation. The increased plan complexity might lead to challenges in radiation delivery, but no delivery errors could be detected in the verification measurements. More MUs might also lead to more head scatter and higher peripheral doses.

A commercially available a posteriori MCO approach [15] has also demonstrated its potential in WPRT-VMAT planning [16]. Knowledge-based treatment planning strategies have also emerged, which use a model created from a library of approved plans to optimize dose distributions for a new patient [17]. However, these approaches still require manual interaction or the establishment of a library of high-quality treatment plans. One limitation of this study is that the Pareto-optimal pre-optimizer (with subsequent reconstruction in Monaco) in autoVMAT was not compared to a manual multi-criterial optimizer [6, 15], but rather to current standard clinical practice planning.

Precise concurrent targeting of the prostate and the pelvic lymph nodes with radiation can be challenging, as both target volumes can move independently. The lymph nodes remain relatively fixed to the bony anatomy, while the prostate may shift more than 15 mm [18, 19] in position with variable bladder and rectum filling. Therefore, adaptive RT (ART) has been proposed for WPRT by employing offline replanning or preplanned plan libraries [20–22]. These approaches are rarely clinically realized due to the high workload. With the demonstrated efficiency increase achieved through autoplanning, multiple VMAT plannings and the implementation of ART for WPRT may be feasible.

Another advantage of automated planning is the absence of inter- and intra-planner variations, and higher a homogeneity in plan quality. This may lead to more consistent outcomes in treatment planning studies and clinical trials.

A limitation of the presented (clinical) planning workflow is that the boost plan is created in the initial planning step and possible systematic changes in anatomy over the first weeks of treatment are not incorporated.

Hip prostheses are rather common in the prostate cancer patient population, but these patients were not considered in this study. WPRT planning can be very complex in the presence of hip implants. Voet et al. [23] investigated automated planning for treatment of prostate plus seminal vesicles (no nodes), where automated beam angle optimization was also considered.

Elekta AB is currently working on the integration of Erasmus-iCycle into their commercial Monaco TPS.

## Conclusion

Fully automated VMAT planning of whole-pelvic prostate treatments with large and complex target volumes created dosimetrically superior treatment plans compared to plans generated manually by experts. The autoVMAT plans were favored by the radiation oncologist in 90% of patients and the planning workload was considerably reduced.

## Caption Electronic Supplementary Material

Details about automated VMAT planning with Erasmus-iCycle/Monaco and manual VMAT planning with Monaco
