# Supplementary material for: Automated volumetric modulated arc therapy planning for whole pelvic prostate radiotherapy
Source: Strahlenther Onkol. 2017 Dec 21;194(4):333–42. doi: 10.1007/s00066-017-1246-2 (PMC5869893; doi:10.1007/s00066-017-1246-2)
Supplement: Supplementary file 1 — Details about automated VMAT planning with Erasmus-iCycle/Monaco and manual VMAT planning with Monaco [file 66_2017_1246_MOESM1_ESM.pdf]

Supplementary material for:

Automated VMAT planning for sequential-boost whole pelvic prostate radiotherapy

Strahlentherapie und Onkologie

By [1]M. Buschmann*, [2]A. Sharfo, [2]J. Penninkhof, [1]Y. Seppenwoolde, [1]G. Goldner, [1]D. Georg, [2]S. Breedveld and [2]B. Heijmen

*corresponding author: martin.buschmann@akhwien.at

[1]Department of Radiation Oncology and Christian Doppler Laboratory for Medical Radiation Research for Radiation Oncology Medical University of Vienna/AKH Wien, Vienna, Austria

[2]Department of Radiation Oncology, Erasmus MC Cancer Institute, Rotterdam, The Netherlands

Automated VMAT planning with Erasmus-iCycle/Monaco:

Automated treatment plan generation with Erasmus-iCycle for an individual patient is based on a site-specific ‘wish-list’ containing the clinical plan objectives with ascribed priorities and hard constraints to be strictly obeyed. The wish-list used for the whole pelvis contains constraints to control maximum and mean dose in the targets and OARs, and dose fall-off outside the PTV. PTV coverage and its homogeneous dose distribution are optimized using the Logarithmic Tumor Control Probability (LTCP) function [1] with the highest priority (priorities 1 and 2), followed by a shell around the PTV to create a conformal dose distribution (priority 3). Equivalent uniform dose (EUD) [2] objectives with volume effect parameter (k = 12 and 4) focusing on reduction of the high and midrange dose in the rectum and bladder, respectively (priorities 4 and 5). Two more shells around the PTV (priority 6 in Table 2a) were defined to make a conformal dose distribution. To reduce mid- to high-range dose in the bowel bag another EUD with (k = 8) was defined (priority 7). A skin ring of 2 cm wide, from the body contour towards the patient’s internal was defined to control entrance doses (priority 8). Mean dose in the rectum, bladder and bowel bag were then reduced with priorities 9,10, and 11. For the left and right femoral heads, EUD objectives were used as shown in Table 2a. After plan optimization in Erasmus-iCycle, a template containing the achieved results for constraints and objectives is automatically created and fed into the clinical TPS (Monaco v5.11, Elekta AB, Sweden) to generate, without planner interaction, clinically deliverable dual-arc VMAT plans.

The following segmentation settings in Monaco were used and kept constant for all automated and manual plan in both planning phases:
fluence smoothing = medium
max # of control points per arc = 230
min. segment width = 1 cm
sector increment = 30°

[1] Alber M, Reemtsen R (2007) Intensity modulated radiotherapy treatment planning by use of a barrier-penalty multiplier method. Opt Methods Softw 22:391-411.

[2] Niemierko A (1997) Reporting and analyzing dose distributions: a concept of equivalent uniform dose. Med Phys 24:103-10.

Manual VMAT planning with Monaco:

In the following tables the cost function templates for manual planning in Monaco are presented. Plan optimizations were initiated with these population-based templates and in the next iterative steps functions and parameters were manually tweaked to obtain the best possible dose distributions.

Table 1S: Applied Monaco template for manual VMAT plan generation for phase 1 (i.e., whole pelvis) plan.

Volume	Type	Dose level	Parameters	Shrink margin
**PTV_P**	Target EUD	60 Gy	Cell sensitivity: 0.5	
	Quadratic overdose	61 Gy	RMS Dose excess: 0.17 Gy	
	Target penalty	58.8 Gy	Min. volume: 99%	
**PTV_{LN}**	Target EUD	50 Gy	Cell sensitivity: 0.5	
	Quadratic overdose	51 Gy	RMS Dose excess: 0.3 Gy	0.5 cm
	Quadratic overdose	52 Gy	RMS Dose excess: 0.1 Gy	0.5 cm
	Target penalty	49.5 Gy	Min. volume: 99%	
**Rectum**	Serial	46 Gy	PLE 15	
	Serial	32 Gy	PLE 10	1 cm
**Bladder**	Serial	41 Gy	PLE 10	
	Serial	32 Gy	PLE 6	1 cm
**Bowel**	Serial	34 Gy	PLE 10	
	Quadratic overdose	50 Gy	RMS Dose excess: 0.1 Gy	
**Le.+Ri. Femoral Head**	Serial	24 Gy	PLE 6	
**Body**	Quadratic overdose	53 Gy	RMS Dose excess: 0.08 Gy	
	Quadratic overdose	41 Gy	RMS Dose excess: 0.17 Gy	0.6 cm
	Quadratic overdose	28 Gy	RMS Dose excess: 0.7 Gy	1.5 cm

Abbreviations: PTV_P = planning target volume of the prostate, PTV_{LN} = planning target volume of the lymph nodes, EUD = Equivalent Uniform Dose, PLE = Power Law Exponent, RMS = Root Mean Square.

Table 2S: Applied Monaco template for manual VMAT plan generation for phase 2 (i.e., boost) plan.

Volume	Type	Dose level	Parameter	Shrink margin
**PTV_P**	Target EUD	13 Gy	Cell sensitivity: 0.5	
	Quadratic overdose	13.1 Gy	RMS Dose excess: 0.3 Gy	
	Target penalty	12.9 Gy	Min. volume: 98%	
**Rectum**	Serial	10 Gy	PLE 15	
	Serial	5 Gy	PLE 10	1 cm
**Bladder**	Serial	9 Gy	PLE 10	
**Le.+Ri. Femoral Head**	Serial	3 Gy	PLE 6	
**Body**	Quadratic overdose	10 Gy	RMS Dose excess: 0.16 Gy	
	Quadratic overdose	5 Gy	RMS Dose excess: 0.3 Gy	1.5 cm

Abbreviations: PTV_P = planning target volume of the prostate, EUD = Equivalent Uniform Dose, PLE = Power Law Exponent, RMS = Root Mean Square.
